# Blood‐Coagulation‐Inspired Dynamic Bridging Strategy for the Fabrication of Multiscale‐Assembled Hierarchical Porous Material

**DOI:** 10.1002/advs.202204702

**Published:** 2022-11-22

**Authors:** Lin Zhang, Yuxin Sun, Li Peng, Wenzhang Fang, Qiao Huang, Jie Zhang, Ziyan Zhang, Hang Li, Yingjun Liu, Yibin Ying, Yingchun Fu

**Affiliations:** ^1^ College of Biosystems Engineering and Food Science Zhejiang University Hangzhou 310058 China; ^2^ International Research Center for X Polymers Department of Polymer Science and Engineering Zhejiang University Hangzhou 310027 China

**Keywords:** adsorption, blood clotting, fibrin, hierarchical porosity, multi‐level design

## Abstract

Porous materials, from macroscopic bulk materials (MBs) with (sub‐)millimeter‐scale pores to tiny particles (TPs) with (sub‐)nanometer‐scale pores, have attracted ever‐growing interest in various fields. However, the integration of multi‐scale pores in one composite is promising but challenging, owing to the considerable gap in the scale of the pores. Inspired by blood coagulation, a fibrin‐based dynamic bridging strategy is developed to fabricate a multiscale‐assembled hierarchical porous material (MHPM), in which fibrin formed as the sub‐framework for the weaving‐narrow of MBs and the enwinding‐load of TPs. The bio‐polymerization nature makes the fabrication rapid, facile, and universal for the customizable integration of seven kinds of TPs and four kinds of MBs. Besides, the integration is controllable with high load capacity of TPs and is stable against external shock forces. The unique multi‐level structure endows the MHPM with large and accessible surface area, and efficient mass transfer pathways, synergistically leading to high adsorption capacity and rapid kinetics in multiple adsorption models. This work suggests a strategy for the rational multi‐level design and fabrication of hierarchical porous architectures.

## Graphical abstract

A bio‐inspired fibrin‐based strategy is reported to integrate macro‐porous bulk materials and nano‐porous particles into a multiscale‐assembled hierarchical porous material. The unique multi‐level porous structure endows the material with the compatibility of large and accessible surface area and efficient mass transfer pathways, synergistically leading to high adsorption capacity and rapid kinetics in multiple adsorption models.

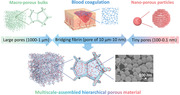

## 1 Introduction

Porous materials have stimulated a great deal of interest in multiple fields, such as energy storage and conversion, environmental remediation, chemical production, biomedicine, and agriculture.^[^ ^1^, ^2^, ^3^, ^4^, ^5^ ^]^ As the pore size scale changes from (sub‐)millimeters to (sub‐)nanometers, the functions and applications of porous materials are extremely diverse. Typically, macroscopic bulk materials (MBs) with (sub‐)millimeter‐scale pores, such as sponges and foams, exhibit rapid mass transfer, excellent mechanical stability, and ease of operation.^[^ ^6^ ^]^ Nevertheless, massive pores also cause low utilization of the inner space and limited specific surface area per unit volume. From another perspective, tiny particles (TPs) with (sub‐)nanometer‐scale pores, such as metal‐organic frameworks (MOFs) and porous graphene, have also drawn increasing attention owing to high specific surface area, abundant active sites, and even confinement effect.^[^ ^1^, ^7^, ^8^, ^9^ ^]^ However, TPs are generally present as brittle and aggregated powders, severely restricting their actual application at scale.^[^ ^3^, ^10^, ^11^, ^12^ ^]^ Therefore, to integrate the merits of diversified pore‐size‐based advantages and operation convenience, an increasing number of efforts have been made to combine MBs and TPs into one hierarchical porous macroscopic material.^[^ ^13^, ^14^ ^]^ Particularly, the fabrication method that enables synergy for application is of considerable interest.

Currently, processing TPs into MBs has been successfully developed to prepare various aerogels/foams with high surface area.^[^ ^15^, ^16^, ^17^ ^]^ However, the products generally are constrained by limited diffusion kinetics and mechanical stability, due to the ill‐defined pore structure and the aggregation of the TPs. An alternative strategy is the loading of TPs on the preformed MBs via in‐situ growth^[^ ^18^, ^19^ ^]^ or post‐modification.^[^ ^20^, ^21^ ^]^ The obtained composites inherit the characteristics of TPs and MBs, showing significant prospects in various fields, such as adsorption and separation, catalysis, and adsorption.^[^ ^1^, ^18^ ^]^ Despite remarkable progress, only the framework of the MBs has been utilized while the pores remain unexplored, leading to limited loads of the TPs. This could be one of the most significant obstacles in exploring the functions and improving the performance. Moreover, most methods require time‐consuming and complicated procedures of surface treatments, growth, or linking.^[^ ^10^ ^]^ Therefore, a new strategy to develop a higher‐level hierarchical structure in a simple manner is highly desirable yet challenging. A major hurdle comes from the diversity of pore structures and the vast gap between these cross‐scale pores (around five orders of magnitude from nanometer to millimeter). In this regard, finding a porous and adhesive sub‐framework to bridge MBs and TPs is the most promising solution.

**Figure 1:**
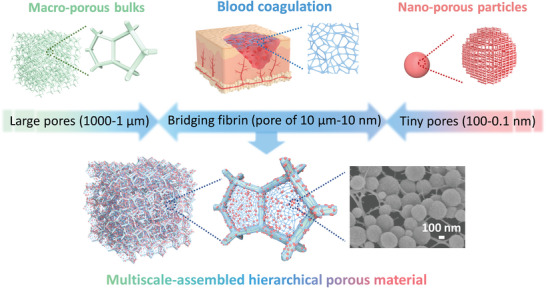
Schematic of the blood‐coagulation‐inspired fibrin‐based dynamic bridging of MBs and TPs into a MHPM.

Nature can provide a rich source of inspiration for materials design and utilization.^[^ ^22^, ^23^, ^24^ ^]^ Blood coagulation is a common but delicate physiological behavior in living systems. In its final stage, fibrinogen (Fg) polymerizes into fibrin to form a clot with blood cells, thereby sealing and facilitating the healing of wounds with diameters from micrometers to even centimeters.^[^ ^25^ ^]^ Unlike flexible polymers, fibrin presents as a robust fibrous network with pores from ∼10 nm to ∼10 µm.^[^ ^26^, ^27^ ^]^ Together with inherent adhesivity, it, as a bio‐glue, has shown significant prospects in biomedical engineering.^[^ ^28^, ^29^, ^30^ ^]^ In this study, we propose a fibrin‐based dynamic bridging strategy to integrate MBs and TPs into a multiscale‐assembled hierarchical porous material (MHPM), in which fibrin serves as the bridging sub‐framework for the weaving‐narrow of the MBs and the enwinding‐load of the TPs (**Figure** **1**). In sharp contrast to previous structures of hierarchical two‐level pore bridging via a direct combination of MBs and TPs, the MHPM achieves the integration of multi‐scale pores thereby achieving compatibility of high surface area, rapid mass transfer, and handleability characteristics for the application. The integration is stable and controllable with a high load capacity of TPs (the content of the loaded MOFs is up to ∼41 wt.%). Moreover, the method described in this paper is readily adaptable to integrate random combinations of various MBs and TPs with different pore structures and morphologies. As a proof‐of‐principle application, the MHPM demonstrates superior adsorption capacity toward targets in solution under all models (static adsorption, dynamic filtration, as well as simultaneous adsorption of multiple targets). Remarkably, the MHPM exhibits excellent potential for practical applications, as proved by the powerful adsorption capability toward single or multiple targets in real‐world samples. This work will provide new horizons and inspirations for the multi‐level design of hierarchically porous materials for energy, environmental, and agricultural applications.

## 2 Results and Discussion

**Figure 2:**
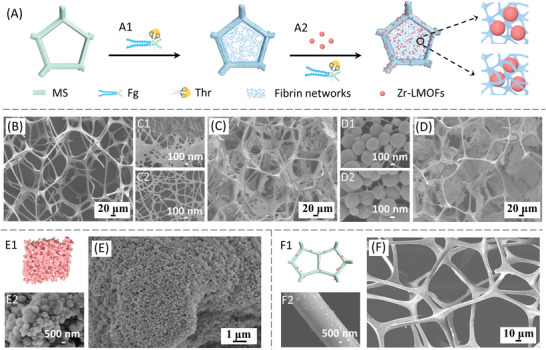
The fibrin‐based dynamic bridging strategy. A) Schematic of the fabrication process. A1 and A2 represent the bridging of fibrin towards the MS and Zr‐LMOFs, respectively. SEM images of B) pure MS, fibrin C1) on the framework and C2) in the pores in C) F@MS, Zr‐LMOFs D1) captured and D2) enwound by fibrin nanofibers in D) MOF‐F@MS. E1) Schematic and E) SEM image of MOF‐assembly. E2 is a magnified SEM image to present the aggregation of Zr‐LMOFs with unclear pore structure. F1) Schematic and F) SEM image of MOF@MS. F2 is a magnified SEM image to present few aggregated Zr‐LMOFs on the framework.

As a proof of concept, we selected melamine sponge (MS) and zirconium‐based luminescent MOFs (Zr‐LMOFs) as the models of MBs and TPs, respectively. MS is a typically commercialized sponge with sub‐millimeter pores, while microporous Zr‐LMOF is a promising material for adsorption and sensing (Figure S1, Supporting Information).^[^ ^31^ ^]^ In a typical fabrication process, solutions of Fg and thrombin (Thr) were respectively added to the MS, wherein fibrin fibers formed in situ and accumulated on the framework or inside the pores, and gradually interconnected to establish a primary network in the MS (**Figure** **2**A1). Within the subsequent addition of a Zr‐LMOF suspension (Figure 2A2), Zr‐LMOFs would be rapidly captured and adsorbed to the fibrin networks, owing to the interactions between a large number of amino acid residues in fibrin and zirconium (Zr^4+^) clusters and aromatic ligands of Zr‐LMOFs. Meanwhile, within the residual Fg, Thr, and fibrin relatives, new fibrin fibers would continuously form around Zr‐LMOFs and further interconnect with fibrin networks, realizing the integration of MS and Zr‐LMOFs.

Scanning electron micrograph (SEM) images were collected to illustrate the strategy (Figure 2A) and the microstructure. As shown in Figure 2B, the pristine MS exhibited an open pore structure with a pore size of 40—200 µm. After a 1 min reaction of Fg and Thr, a few fragmentary fibers stretched across the MS pores, and the surfaces of the MS framework were coated by fibrin fibers with a diameter of ∼100 nm (Figure S2A, Supporting Information). As the bio‐polymerization proceeded, more fibrin fibers formed, and the pores of the MS began to be bridged (Figure S2B, Supporting Information). Particularly in the region near the framework, porous fibrin films appeared and interconnected with the fibers that densely coating the framework (Figure 2C1), which acted as anchors for the further expansion of fibrin bridging networks. After 7 min, the bridging continued as the area of the fibrous film increased, most notably in the center (Figure 2C2), successfully forming the sub‐framework with (sub)micrometer diameter pores inside the sub‐millimeter pores of the MS (F@MS, Figure 2C). These images illustrate the dynamic bridging process of fibrin, weaving from the MS framework to the center. The dense distribution of fibrin near the framework would strengthen its connection and mechanical stability, providing a stable matrix for the following load of Zr‐LMOFs. Coating polymers on MBs have been widely adopted for the functionality offered. However, general methods have only achieved the coating of the frameworks^[^ ^19^ ^]^; this is mainly because the building units of polymer monomers are too small, and the chains too flexible to bridge the large pores. On the contrary, Fg is a rigid rod‐shaped protein with a length of ∼450 Å. It can expand via linking end‐to‐end to form fibrin fibers,^[^ ^32^ ^]^ making itself a perfect unit to construct a network across the pore.

Further, with the addition of Zr‐LMOFs, many spherical particles were directly attached to or inserted into the fibrous networks, and dispersed uniformly without apparent aggregation (Figure 2D1). Interestingly, some Zr‐LMOFs were enwound by a few nanofibers incorporated in the networks (Figure 2D2). These nanofibers are the products of the continuous formation of fibrin after adding Zr‐LMOFs, which would not only benefit to the exposure of external surfaces of the MOF crystals, but also increase the amount and stability of the Zr‐LMOFs. In this regard, a MHPM of Zr‐LMOFs‐fibrin@MS (MOF‐F@MS, Figure 2D) was achieved based on the bridging of fibrin networks. Namely, the MS serves as the primary structure for robust support. Fibrin serves as the second‐level bridging framework for the load of Zr‐LMOFs, and the reservation of mass transfer pathways. Zr‐LMOFs make up the third‐level structure and act as the functional elements with large specific surface area and abundant active sites for applications. In contrast, unclear pore structures and aggregation of TPs were found in the assembled material obtained by high‐speed centrifugation, which illustrated the difficulty of processing TPs into MBs (the product was defined as MOF‐assembly, Figure 2E). As for the post‐modification methods for the integration, conventional methods generally suffer from limited space utilization of MBs and a lack of control of the organization of TPs.^[^ ^31^, ^33^, ^34^ ^]^ As shown in Figure 2F, through in‐situ solvothermal growth of Zr‐LMOFs on the MS (the product was defined as MOF@MS), a few aggregated particles were randomly coated on the framework, leaving the large inner space of pores unexplored.

**Figure 3:**
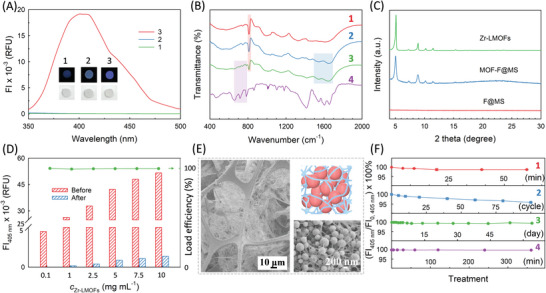
Characterization of the integration and the MHPMs. A) Fluorescent emission spectra of 1) MS, 2) F@MS and 3) MOF‐F@MS. Insets are the corresponding photographs under (top) ultraviolet and (bottom) daylight. B) FT‐IR spectra of 1) MS, 2) F@MS, 3) MOF‐F@MS and 4) Zr‐LMOFs. C) XRD patterns. D) FI_405nm_ of the Zr‐LMOFs suspensions with different concentrations before and after the loading onto MOF‐F@MS, and the calculated load efficiencies of Zr‐LMOFs. E) SEM images and schematic of MOF‐F@MS prepared within a high concentration of Zr‐LMOFs (10 mg mL^−1^). F) Fluorescent changes of MOF‐F@MS before (FI_0_) and after (FI) external shock forces of 1) vortex, 2) pressing, 3) soaking in solution, and 4) ultrasound. The experiment was repeated three times.

The rapidity of blood coagulation, essential to survival, comes from the rapid formation of fibrin networks to bridge the wound. Hence, the rapid loading of TPs was expected. The fluorescence intensities (FI) of the solutions extracted from the MOF‐F@MS with different fabrication times were monitored. As shown in Figure S3, Supporting Information, fluorescence peaks of Zr‐LMOFs completely disappeared in all solution samples. These indicated all MOFs have been entrapped in the MS in less than 0.25 min, which sharply contrasts with conventional methods that generally require several hours or even days.^[^ ^17^, ^33^ ^]^ MOF‐F@MS exhibits an apparent blue fluorescence with a fluorescent emission peak around 405 nm, indicating the successful loading and the reserved fluoresent charactertics of Zr‐LMOFs (**Figure** **3**A). This is attributed to the excellent formability of fibrin deriving from a highly‐efficient biological polymerization mechanism and a superior adhesive capacity. Besides, this simple and rapid manner allows the fabrication to have excellent controllability and reproducibility (Figure S4, Supporting Information).

Fourier‐transform infrared spectroscopy (FT‐IR) spectra were collected to evaluate the integration of different components in the MOF‐F@MS (Figure 3B). MS exhibited a clear characteristic peak at ∼812 cm^−1^ that is indicative of the triazine ring bending (red area on line 1).^[^ ^35^ ^]^ In the spectrum of F@MS (line 2), two new peaks appeared 1542 and 1652 cm^−1^ ascribed to amide I and II of protein (blue area), demonstrating fibrin formation in the MS.^[^ ^30^ ^]^ For MOF‐F@MS (line 3), besides the above peaks, parts of characteristic peaks of Zr‐LMOFs^[^ ^36^ ^]^ (purple area) could be observed, such as the vibration peaks of a Zr–O bond at 667 cm^−1^ and a C–H substituted benzene ring at 782 cm^−1^, demonstrating the successful fabrication of MOF‐F@MS. Further, powder X‐ray diffraction (XRD) was used to confirm the crystal structure. As shown in Figure 3C, strong diffraction peaks at 2*θ* values of 5.05, 7.31, 8.87, 10.15, and 11.42° that indicate Zr‐LMOFs^[^ ^31^ ^]^ showed up in MOF‐F@MS, indicating the highly crystalline structure of the loaded Zr‐LMOFs. Figure S5, Supporting Information, gives the nitrogen adsorption‐desorption isotherms of different sponges. MS within sub‐millimeter pores presented a negligible Brunauer–Emmett–Teller (BET) surface area. After the formation of fibrin network across the MS, the BET surface area of F@MS increased to 7.23 m^2^ g^−1^. As for MOF‐F@MS, a different type‐IV adsorption‐desorption isotherm was found, revealing the presence of mesopore and micropore in the MHPMs. The BET surface area of MOF‐F@MS was dramatically increased to 197.62 m^2^ g^−1^, which was largely attributed to the presence of microporous Zr‐LMOFs.

The load capacity of Zr‐LMOFs in MOF‐F@MS was investigated by fluorescent quantification of the residual MOFs in solution (Figure 3D and Figure S6, Supporting Information). The load efficiencies were estimated to be 99.9%, 98.8%, 99.2%, 99.4%, 99.0 %, 99.3% and 98.2% with different initial concentrations of Zr‐LMOFs of 0.1, 1, 2.5, 5, 7.5, 10, and 15 mg mL^−1^, respectively, which indicates almost all MOFs were loaded. Furthermore, the strong load capability also allowed a high degree of control on the amount of Zr‐LMOFs in MOF‐F@MS, through adjusting the concentration of the added Zr‐LMOFs suspension in a wide range from 0.1 to 15 mg mL^−1^. This is not simply achieved by other methods, regardless of the chemical conversion or physical processing. The maximum load content of Zr‐LMOFs achieved was ∼41 wt.% in MOF‐F@MS (Figure S6B, Supporting Information). These performances benefit from the unique integration strategy and sophisticated architecture. Basically, the protein nature of Fg and fibrin with abundant functional groups ensures the entrapment of a large amount of Zr‐LMOFs. On the other hand, as shown in Figure 3E, MOF‐F@MS with a large content of Zr‐LMOFs also presented as a MHPM, in which several layers of Zr‐LMOFs organized by fibrin nanofibers were found in the pores of the MS. This interesting structure formed from the dynamic bridging assembly, specifically, the fiber that enwound on the first layer of Zr‐LMOFs continuously attracted other Zr‐LMOFs to stack, and then, these Zr‐LMOFs in turn acted as the anchors for the further formation of new fibrin nanofibers. With each cycle, more Zr‐LMOFs were loaded with each layer added. Meanwhile, due to the mismatched stacking and the interval of each layer, they were still relatively porous, maintaining the mass transfer pathways but significantly increasing the specific surface area.

In addition to the successful integration, structural integrity is also a key factor in evaluating the application potential. Consequently, the stabilities of MOF‐F@MS under various rigorous conditions have been investigated in detail. Under SEM characterization, all samples after treatments were present as typical MHPMs, illustrating a structural reliability against external shock forces of vortex, pressing, soaking, and ultrasound (Figure S7, Supporting Information). Furthermore, the stability was quantified by comparing the FI of the incubation solution before and after the treatment (Figure S6‐A, Supporting Information). As shown in Figure 3F, the remained amounts of the loaded Zr‐LMOFs were calculated to be (98.7 ± 0.2)% after being vortexed for 60 min, (95.7 ± 0.2)% after a 100‐cycle pressing, (99.6 ± 0.1)% after a 64‐day soaking, and (99.9 ± 0.1)% after a 360 min ultrasound, respectively. All results above prove the extraordinary integrity and stability of MOF‐F@MS, which were attributed to the inherent strong adhesion of fibrin to both MS and Zr‐LMOFs. More importantly, the fibrin nanofibers enwinding on the particles could protect Zr‐LMOFs against external shock force.

**Figure 4:**
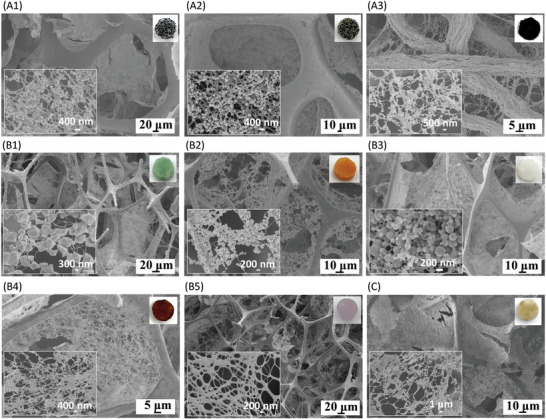
The universality of the strategy to integrate different MBs and TPs into MHPMs. SEM images of A1) MOF‐F@polyurethane‐foam, A2) MOF‐F@nickel‐mesh and A3) MOF‐F@graphene‐fabric, B1) MIL‐101(Cr)‐F@MS, B2) MIL‐100(Fe)‐F@MS, B3) mesoporous‐silica‐F@MS, B4) MNPs‐F@MS and B5) AuNPs‐F@MS, C) Zr‐LMOFs‐MIL‐100(Cr)‐MIL‐100(Fe)‐F@MS. Insets are the SEM images inside the pore of the MBs (bottom) and the photographs of the MHPMs (top).

To prove the universality of the strategy, three kinds of MBs (polyurethane, nickel sponges, and graphene fabric) were fabricated with Zr‐LMOFs (**Figure** **4**A1 to 4A3). All the obtained products exhibited as MHPMs, in which fibrin networks not only adhered or intercrossed firmly with the framework, but also loaded many Zr‐LMOFs, demonstrating the powerful bridging ability of fibrin across various pore structures, from curved circles to linear triangles. Five kinds of representative TPs with different sizes, morphologies, components, and properties: MIL‐101(Cr), MIL‐100(Fe), mesoporous silica, magnetic beads (MNPs), and gold nanoparticles (AuNPs), were also successfully integrated with the MS (Figure 4B1 to 4B5). The multiscale hierarchical porous structure and load capacity of TPs remained in the products, which benefited from the universal loading capability of fibrin and its unique formation process. Additionally, different kinds of TPs (Zr‐LMOFs, MIL‐100(Cr), and MIL‐100(Fe)) were successfully integrated into one MHPM with uniform distribution of TPs (Figure 4C). This is rarely achieved through conventional methods, or complicated multi‐step procedures are required, due to the limited formability of the binders. The integration of diversified components into one material could create unprecedented performance and functions that surpass individual components.

**Figure 5:**
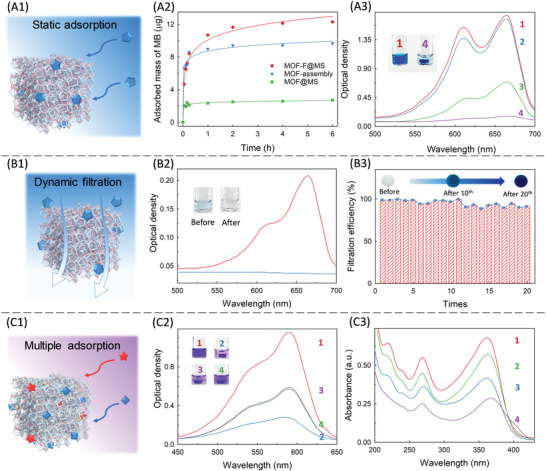
Adsorption applications of MHPMs towards targets in solution. A) Static adsorption: A1) schematic diagram, A2) time‐dependent adsorption curves of different adsorbents towards methylene blue, A3) UV‐Vis spectra of the methylene blue solution 1) before and after the incubation within 2) MOF‐assembly, 3) MOF@MS and 4) MOF‐F@MS. The experiment was repeated three times. Insets are the photographs of methylene blue solution 1) before and 4) after the incubation with MOF‐F@MS. B) Dynamic filtration: B1) schematic diagram, B2) UV‐Vis spectra and photographs (insets) of methylene blue solution before (red line) and after (blue line) the filtration through MOF‐F@MS, B3) the calculated adsorption efficiency in 20 times. Insets are photographs of MOF‐F@MS during the filtration. C) Versatile adsorbents for synergistic and complementary adsorption: C1) schematic diagram, C2) UV‐Vis spectra of CV solution 1) before and after the incubation within 2) Zr‐Z67‐F@MS, 3) MOF‐F@MS, and 4) Z67‐F@MS, insets are the corresponding photographs, C3) UV‐Vis spectra of the mixture solution of TC and AFB_1_ before 1) and after the incubation of 2) Z67‐F@MS, 3) MOF‐F@MS and 4) Zr‐Z67‐F@MS.

An impressive advantage of a MHPM is the compatibility of the large specific surface area and efficient mass transfer, which are favorable for adsorption‐relevant applications. As a proof‐of‐principle application, the adsorption of the MHPM toward targets in solution was systematically investigated and compared (**Figure** **5**). Firstly, the static adsorption was evaluated (Figure 5A), using methylene blue as the target model. To directly assess the remaining adsorption capacity of Zr‐LMOFs in the assemblies, adsorption kinetics were recorded (Figure 5A2). The adsorption rates of MOF‐F@MS, MOF@MS, and MOF‐assembly towards methylene blue were comparable, while the equilibrium adsorption capacity of MOF‐F@MS (12.42 ± 0.49 µg) was higher than that of MOF@MS (2.73 ± 0.53 µg) and MOF‐assembly (9.65 ± 0.46 µg). The time‐dependence curve of the adsorption of MOF‐F@MS fitted well with the pseudo‐second‐order kinetic model (Figure S8, Supporting Information), which implied that the adsorption performance was dominated mainly by intraparticle diffusion.^[^ ^15^, ^16^ ^]^ Therefore, our strategy and the corresponding structure did not hinder the accessibility of the micropores of Zr‐LMOFs. Moreover, the adsorption efficiency of MOF‐F@MS towards a high concentration of methylene blue (150 mg L^−1^) was as high as 95.7%, while MOF@MS and MOF‐assembly exhibited low efficiencies of 9.1% and 64.7%, respectively (Figure 5A3). The methylene blue solution faded into light blue, while MOF‐F@MS showed as the blue sponge within the adsorption (inset). The maximum adsorption capacity of MOF‐F@MS was estimated to be 127.6 mg g^−1^, which is higher than other materials in previous reports.^[^ ^37^, ^38^, ^39^, ^40^, ^41^, ^42^ ^]^ The high adsorption rate and high capacity of MOF‐F@MS benefit from the dispersion of Zr‐LMOFs particles and the rapid mass transfer pathways in the MHPM. Additionally, after the static incubation, the MOF‐F@MS was present as the typical MHPM with an ultralow loss ratio of Zr‐LMOFs (<0.01% according to fluorescent quantitation) (Figures S9 and S10, Supporting Information). The adsorption capability of the MOF‐F@MS remained high even after the rigorous treatments of the vortex, pressing, soaking, and ultrasound (Figure S11, Supporting Information). These indicate the robustness of the MOF‐F@MS for adsorption application.

Dynamic adsorption, such as filtration, is also common in practical applications, which requires high adsorption rate and good robustness.^[^ ^43^ ^]^ Here, the filtration efficiency of MOF‐F@MS was evaluated in a homemade filtration device (Figure 5B1 and Figure S12, Supporting Information). After one‐time filtration for a 400 µL 10 mg L^−1^ methylene blue solution, the blue‐colored solution became colorless, and the adsorption efficiency was measured to be 100% (Figure 5B2). Remarkably, the efficiency remained as high as 91% after 20 times, and the MOF‐F@MS adsorbent remained intact except for the color that changed from white to dark blue (Figure 5B3). Microscopically, MOF‐F@MS still remains the typical multi‐level porous structure (Figure S13, Supporting Information), and few Zr‐LMOFs were detached from the MOF‐F@MS after 20 times filtration (an ultralow ratio of <0.25%, Figure S14, Supporting Information). This demonstrates the excellent efficiency and robustness of the MOF‐F@MS, providing great potential for practical application.

Specifically, beyond a single function, different TPs are also highly demanded to be integrated, thereby synergistically optimizing properties for applications, e.g., simultaneous adsorption of multiple targets for the treatment of household and industrial wastewater, catalysis, and sensing.^[^ ^44^, ^45^ ^]^ However, although with complicated procedures, the obtained composites generally suffer from poor structural integrity, limited load capacity, aggregation, and uneven distribution of TPs. This is mainly due to the diversity of TPs and the poor formability of conventional strategies. More seriously, in some cases, the integration may cause mutual interference of different components rather than synergy. As mentioned, our strategy is highly‐efficient in obtaining a MHPM with a high load and uniform distribution of various TPs. Therefore, for the property and application verification, two TPs (i.e., Zr‐LMOFs and ZIF‐67) were integrated to obtain Zr‐Z67‐F@MS for adsorption (Figure 5C1). Under the same conditions, the adsorption efficiency of MOF‐F@MS, Z67‐F@MS, and Zr‐Z67‐F@MS towards crystal violet (CV) were 39%, 37%, and 71.6%, respectively, proving an apparent synergistic effect of Zr‐LMOFs and ZIF‐67 in the Zr‐Z67‐F@MS (Figure 5C2 and Figure S15, Supporting Information). Moreover, as for the complementary model, different properties and functions of diversified TPs also remained active after the integration, exhibiting excellent performance for the simultaneous adsorption of multiple targets in one mixed solution. As shown in Figure S16, Supporting Information, towards the sole adsorption of tetracycline (TC), Z67‐F@MS exhibited better capacity than MOF‐F@MS, while in the case of aflatoxin B1 (AFB_1_), the adsorption was negligible in Z67‐F@MS. As for the mixture solution of TC and AFB1 (Figure 5C3), the adsorption efficiency of Z67‐F@MS, MOF‐F@MS, and Zr‐Z67‐F@MS were 38.5%, 15.7%, and 59.1%, respectively, indicating a complementary effect without apparent mutual interference between the different TPs. The simultaneous concentration of multiple targets is difficult to achieve by common adsorbents. These results benefit from the versatility of the strategy we have utilized for fabricating customizable MHPMs.

Finally, to assess the feasibility and potential for practical application, the adsorption performances of the MHPMs toward targets that added in real environmental and agricultural samples were tested. The MOF‐F@MS was first examined for the removal of dye in river water, since the removal of chemical pollutants in the environment is essential to sustainability. The excellent adsorption capability of MOF‐F@MS towards methylene blue added in river water was verified (Figure S17A, Supporting Information). Furthermore, MOF‐F@MS‐based filtration was also performed to mimic the treatment of flowing effluents. As displayed in Figure S17B, Supporting Information, MOF‐F@MS exhibited high filtration efficiency of 99.4% even after 5 times, demonstrating its high adsorption capability, anti‐interference ability, and robustness in practical filtration. Besides, as separation and concentration of trace chemical hazards in agricultural samples is important and challenging for food/agro‐product safety detection. Therefore, a simultaneous concentration of TC and AFB1 in the feed sample was conducted on Zr‐Z67‐F@MS adsorbent. Although the feed sample exhibits lots of chemical and biological interferences, the simultaneous concentration efficiency is as high as (87.3 ± 1.5)% (Figure S17D, Supporting Information). This further validates the practicality of MHPMs for the concentration of multiple targets, which is expected to endow the sensors with higher sensitivity and shorter detection times for practical samples.

## 3 Conclusion

In summary, we have developed a facile and highly efficient fibrin‐based dynamic bridging strategy to integrate MBs and TPs into MHPMs. In sharp contrast to previous concepts of hierarchical porosity for integration, fibrin networks served as the bridging sub‐framework for the weaving narrow of MBs and the enwinding load of TPs, thereby achieving the maximum utilization of cross‐scale pores in one material. Taking advantage of the efficient mass transfer routes, abundant binding sites, and good accessibility, the MHPM presented excellent performance in static adsorption, dynamic filtration, as well as simultaneous adsorption of single and multiple targets with clear synergistic and complementary effects observed. These unique MHPMs are promising for the applications of separation, cascade catalysis, and sensing. The proposed dynamic assembly strategy creates a new way to fabricate multi‐functional macroscopic materials.

## Conflict of Interest

The authors declare no conflict of interest.

## Supporting information

Supporting Information

## Data Availability

The data that support the findings of this study are available in the supplementary material of this article.;
