# Supplementary material for: Blood‐Coagulation‐Inspired Dynamic Bridging Strategy for the Fabrication of Multiscale‐Assembled Hierarchical Porous Material
Source: Adv Sci (Weinh). 2022 Nov 22;10(2):2204702. doi: 10.1002/advs.202204702 (PMC9839836; doi:10.1002/advs.202204702)
Supplement: Supplementary file 1 — Supporting Information [file ADVS-10-2204702-s001.pdf]

ADVANCED SCIENCE
Open Access

Supporting Information

for *Adv. Sci.*, DOI 10.1002/advs.202204702

Blood-Coagulation-Inspired Dynamic Bridging Strategy for the Fabrication of Multiscale-Assembled Hierarchical Porous Material

*Lin Zhang, Yuxin Sun, Li Peng, Wenzhang Fang, Qiao Huang, Jie Zhang, Ziyan Zhang, Hang Li, Yingjun Liu, Yibin Ying and Yingchun Fu**

Supporting Information

For

Blood-Coagulation-Inspired Dynamic Bridging Strategy for the Fabrication of Multiscale-Assembled Hierarchical Porous Material

Lin Zhang[a,b], *Yuxin Sun*[a], *Li Peng*[b], *Wenzhang Fang*[b], *Qiao Huang*[a], *Jie Zhang*[a], *Ziyan Zhang*[a], *Hang Li*[b],*Yingjun Liu*[b], *Yibin Ying*[a], *Yingchun Fu*[a,*]

[a] College of Biosystems Engineering and Food Science, Zhejiang University, Hangzhou 310058, China.

[b] International Research Center for X Polymers, Department of Polymer Science and Engineering, Zhejiang University, Hangzhou 310027, China.

*E-mail: ycfu@zju.edu.cn (Yingchun Fu)

Experimental section

Materials. Fg (F8051) was purchased from Beijing Solarbio Science & Technology Co., Ltd.. Thr from human plasma (T6884) was purchased from Sigma-Aldrich. Zirconium chloride ($ZrCl_4$) and 1,2,4,5-Tetra (4-carboxyphenyl) benzene (H_4TCPB) were purchased from Aladdin Industrial Corporation (Shanghai). Benzoic acid, methylene blue (MB), crystal violet (CV), methanol, ethanol, N, N-dimethylformamide (DMF) were obtained from Sinopharm Chemical Reagent Co. Ltd. (Shanghai, China). Glutaraldehyde (GA) and tetracycline (TC) were purchased from Sangon Biotech Co., Ltd. (Shanghai). Aflatoxin B1 (AFB1) was purchased from Bailingwei Technology Co., LTD. (Beijing, China). MS, polyurethane sponge (3 mm), nickel form (3 mm), and mesoporous silica were purchased from local supermarkets. Graphene fabric was produced by Hangzhou Gaoxi Technology Co. Ltd. ZIF-67, MIL-101(Cr), and MIL-100(Fe) were purchased from Chemsoon Co., Ltd. (Shanghai, China). AuNPs and MNPs were prepared according to the typical protocols described in the literature [1]. Phosphate buffered solution (pH 7.0, 0.01 M NaH_2PO_4-Na_2HPO_4 + 0.15 M NaCl) was used for the formation of fibrin. All reagents were of analytical grade and used without further purification. Deionized water (resistivity $\geq$ 18 MΩ cm) was used to prepare aqueous solutions, and all experiments were performed at room temperature.

All real samples, including river water and feed, were obtained locally (Hangzhou, China). The river water was filtrated by 0.22 mm filter membrane for three times, and used as the solvent directly for the adsorption test of MB. As for the pre-treatment of the feed sample, 1 g powder was immersed in 10 mL methanol-water solution (volume ratio = 1:9). After shaking for 5 min, the suspension was centrifugated and then filtrated for three times. 400 μL of the liquid was collected as the solvent for static adsorption test of TC and AFB1.

Characterization. SEM images were collected on a SU 8010 field-emission scanning electron microscope (Hitachi Ltd., Japan). A transmission electron micrograph (TEM) was recorded using a JEM-1010 transmission electron microscope (JEOL Inc., Japan). FT-IR was performed using an AVATAR 370 spectrometer (Thermo Nicolet, USA).

PXRD pattern study was carried out on a D8 ADVANCE spectrometer (Bruker, Germany). UV-Vis and fluorescence spectra were recorded on an Agilent 8453 UV-vis spectrophotometer (Agilent, USA) and a Synergy H1 hybrid multi-mode microplate reader (BioTek Instruments Inc., USA). Noted that the gain value was adjusted appropriately to avoid the overflow of the fluorescent intensity (FI), but was set as te same value in one experiment. Nitrogen adsorption-desorption isotherms and Brunauer-Emmett-Teller (BET) surface area data were measured on Quantachrome Instruments at 77 K (surface area and pore size analyzer NOVA touch LX4).

Synthesis of Zr-LMOFs. Zr-LMOFs were synthesized by dissolving and mixing 0.05 g $ZrCl_4$, 0.07 g H_4TCPB, and 2.7 g benzoic acid in 32 mL DMF. After a solvothermal reaction at 120 ºC for 6 h, white Zr-LMOFs powders were collected by centrifugation and washed with DMF and MeOH, followed by drying at 60 ºC for 12 h.

Fabrication of various MHPMs. All MBs were cut into specific shapes (column shaped; with a height of 5 mm and diameter of 2 mm), then washed with H_2O and ethanol, and dried at 80 ºC. A cell in the transparent 96-well plate was utilized as the container to fabricate one MHPM.

MOF-F@MS was prepared as follows. Typically, 40 µL of 0.75 mg mL^{-1} Fg was added to MS, which was then squeezed and mixed for uniform distribution. Afterward, 5 µL of 100 U mL^{-1} Thr was added, triggering the in-situ polymerization of fibrin in the MS. After a 7 min reaction for the primary fabrication of F@MS, 20 µL of 1 mg mL^{-1} Zr-LMOFs suspension was directly added and mixed with the above system gently to load the Zr-LMOFs. Another 8 min later, 200 µL of 0.1% GA solution was added for the cross-linkage of fibrin. Finally, the MOF-F@MS was taken out and obtained after being washed with water several times. Control experiments of the F@MS were prepared by replacing Zr-LMOFs suspension with water.

Other TPs-F@MS were fabricated according to the above protocol with the replacement of Zr-LMOFs by 1 mg mL^{-1} of the other TPs, including: ZIF-67, MIL-101(Cr), MIL-100(Fe), mesoporous silica, MNPs, and AuNPs. For the preparation of TPs-F@MS with multiple components of TPs, different TPs suspensions (1 mg mL^{-1}) were mixed in equal volumes, sonicated for dispersion. 20 µL of the mixture was added

to the same F@MS system. Other Zr-LMOFs-F@MBs were fabricated with the replacement of specific MBs with the size and volume equal to the MS.

Adsorption evaluation of MOF-F@MS. UV-Vis spectroscopy was utilized to monitor the concentration change of MB, CV, AFB_1 and TC, and the calibration curve was collected firstly for quantification. As for the evaluation of adsorption performance, samples were incubated in the aqueous solutions of specific targets with the supernatants monitored. The adsorption capacity at time *t* of Zr-LMOFs in MOF-F@MS was calculated using the equation: q_t (mg g^{-1}) = $(c_0\text{-}c_t)\cdot V/m_{\text{Zr-LMOFs}}$, where c_0 and c_t are the concentration of the solution before and after the adsorption, respectively, *V* is the volumn of the solution, and *m* is the mass of Zr-LMOFs in the MOF-F@MS. The detailed conditions for different adsorption models are as follow: i) static adsorption of MB: one piece of MOF-F@MS (ca. 0.5 mg) prepared within 1 mg mL^{-1} Zr-LMOFs was immersed in 400 µL of 50 mg mL^{-1} MB for the evaluation of adsorption kinetics. For comparison of adsorption capability, MOF@MS and MOF-assembly prepared with the equal volumn of the MS or the equal mass of the Zr-LMOFs were used. For the adsorption of MB with a high concentration of 150 mg L^{-1}, MOF-F@MS was prepared with 10 mg mL^{-1} Zr-LMOFs; ii) filtration: MOF-F@MS that prepared with 10 mg mL^{-1} Zr-LMOFs was loaded as the adsorbent in the home-made filtration system; III) static adsorption of CV: pieces of MOF-F@MS, Z67-F@MS and Zr-Z-F@MS were prepared with equal contents of MOFs and independently immersed in 400 µL of 100 mg L^{-1} CV, respectively; IV) simultaneous adsorption of TC and AFB_1: pieces of MOF-F@MS, Z67-F@MS and Zr-Z-F@MS were prepared with equal contents of MOFs and immersed in 400 µL of a mixture of AFB1 (12.5 mg L^{-1}) and TC (12.5 mg L^{-1}).

Statistical Analysis. Data are expressed as means ± standard error of the mean, and all statistical analyses were performed using Sigmaplot (Systat Software Inc., USA).

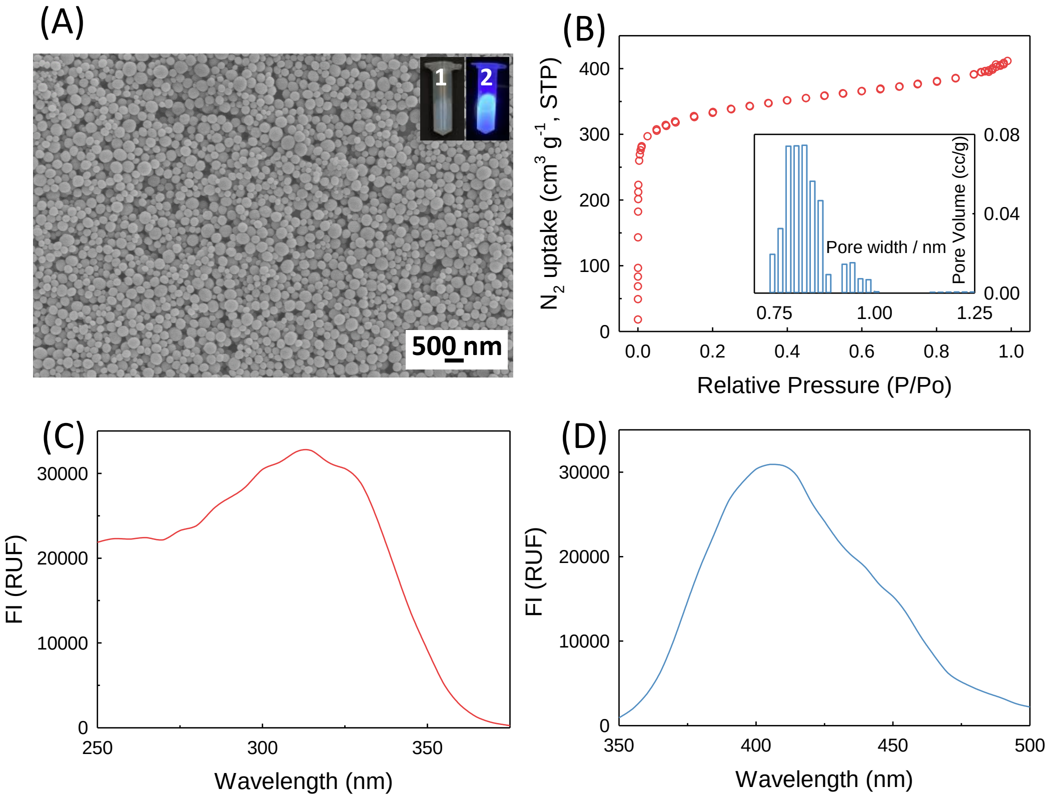

Figure S1. Characterization of Zr-LMOFs. A) SEM image. Insets are the photographs of the suspensions under (1) daylight and (2) ultraviolet. B) N_2 adsorption/desorption isotherm. Inset is the pore size distribution. C) Fluorescent excitation and D) emission spectra.

Zr-LMOFs were present as 100–400 nm spheres with rough surfaces, and possessed high specific surface area (1285.1 m^2 g^{-1}) with approximately 0.85 nm micropores. Zr-LMOFs suspension (in H_2O) presented bright blue fluorescence with the maximum emission wavelength around 405 nm under excitation of 310 nm ultraviolet light.

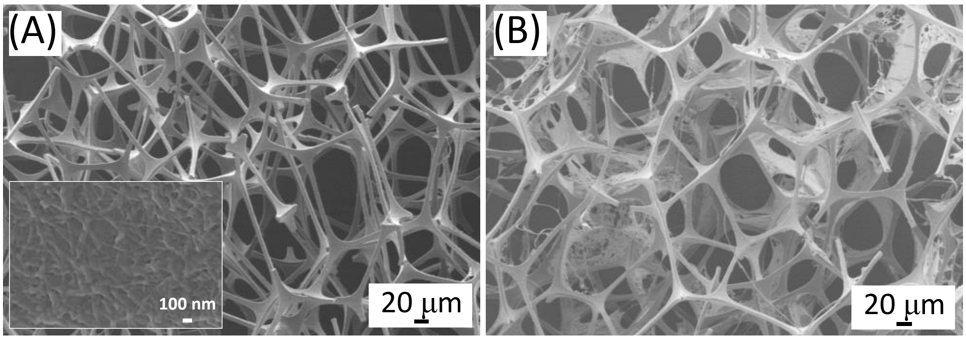

Figure S2. SEM images of F@MS prepared within A) 1 min and B) 3 min reaction for the formation of fibrin in the MS.

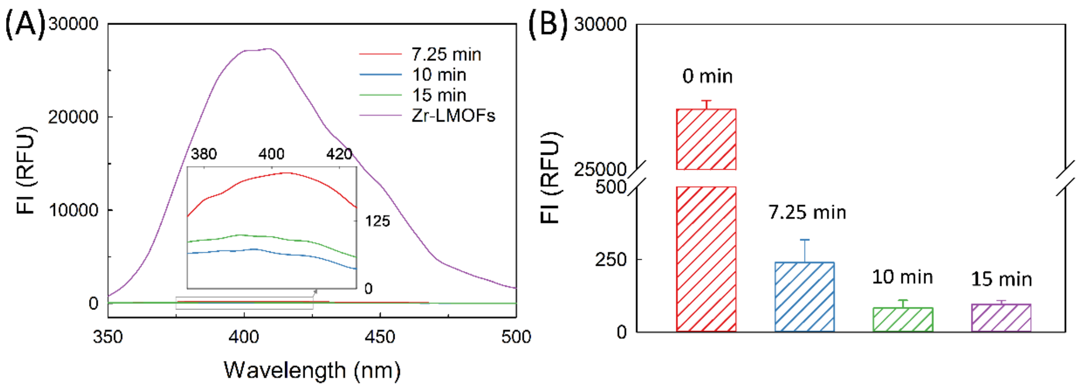

Figure S3. A) Fluorescent spectra and B) FI at 405 nm of the solution extracted from the MOF-F@MS within different reaction times of 0 (Zr-LMOFs suspension), 7.25, 10, 15 min. The values were 27071 ± 301, 239 ± 78, 84 ± 26, and 95 ± 14, respectively. Noted that the first 7 min was used to form primary fibrin networks, and Zr-LMOFs were added at 7 min.

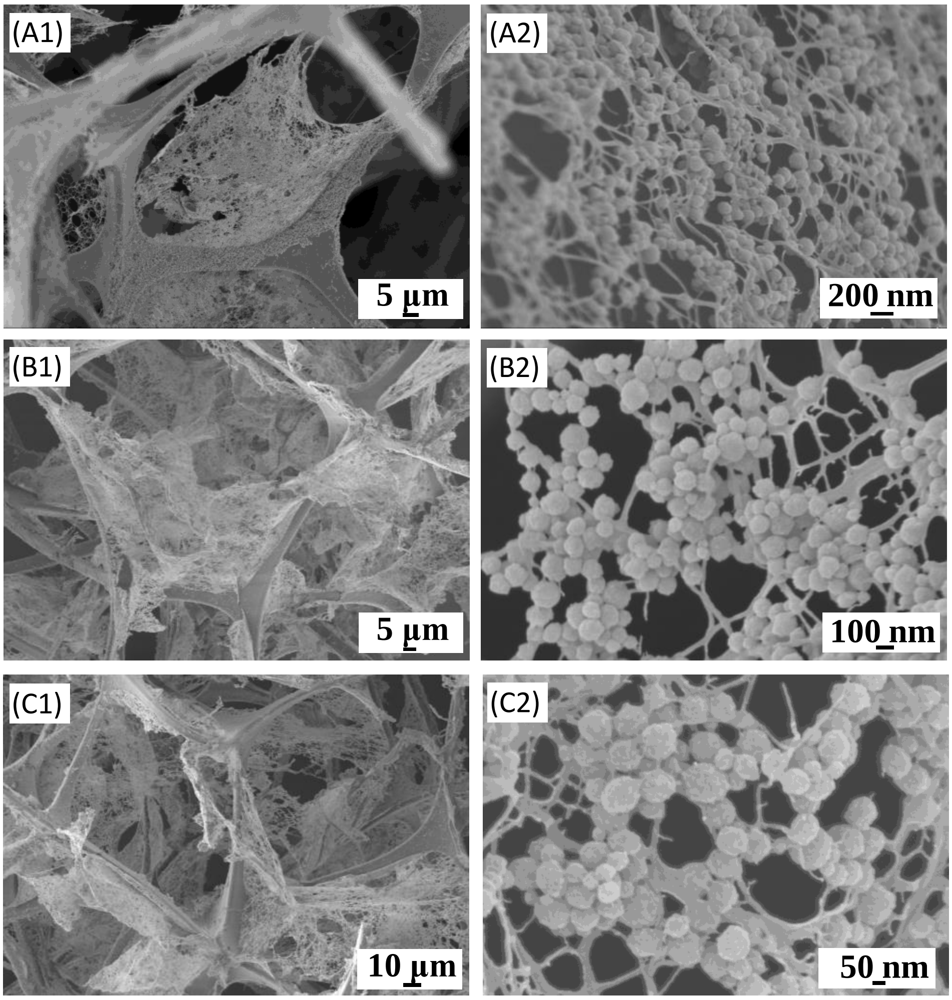

Figure S4. SEM images of MOF-F@MS prepared under the standardized procedure in different batches. All samples of A, B, C present as typical MHPMs, demonstrating the repeatability of the fabrication method. 1 and 2 present the same sample under different magnification scales.

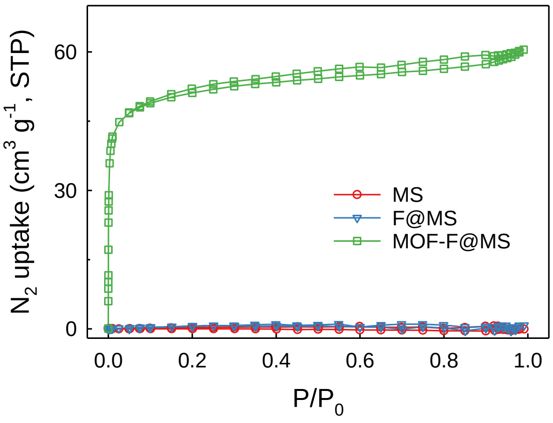

Figure S5. N_2 adsorption-desorption isotherms of MS, F@MS and MOF-F@MS.

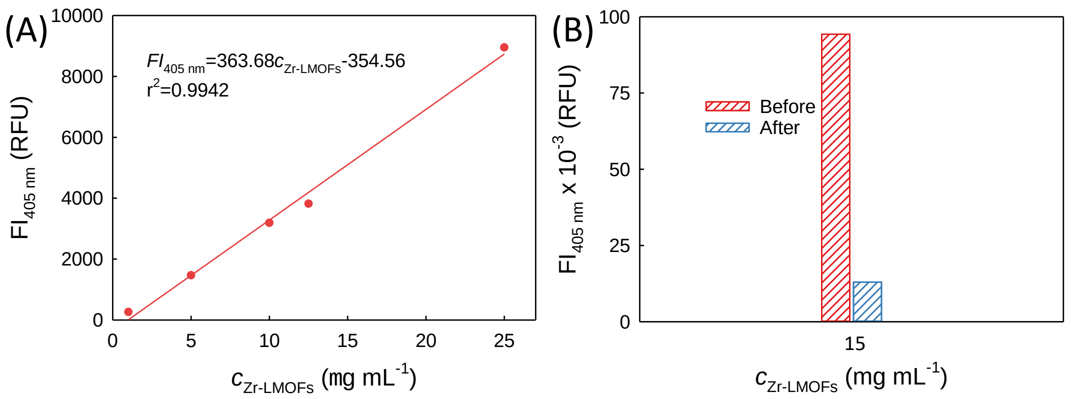

Figure S6. A) Calibration curve for the fluorescent quantification of Zr-LMOFs. B) FI_{405nm} of the Zr-LMOFs suspensions with 15 mg mL^{-1} before and after the load in Zr-F@MS.

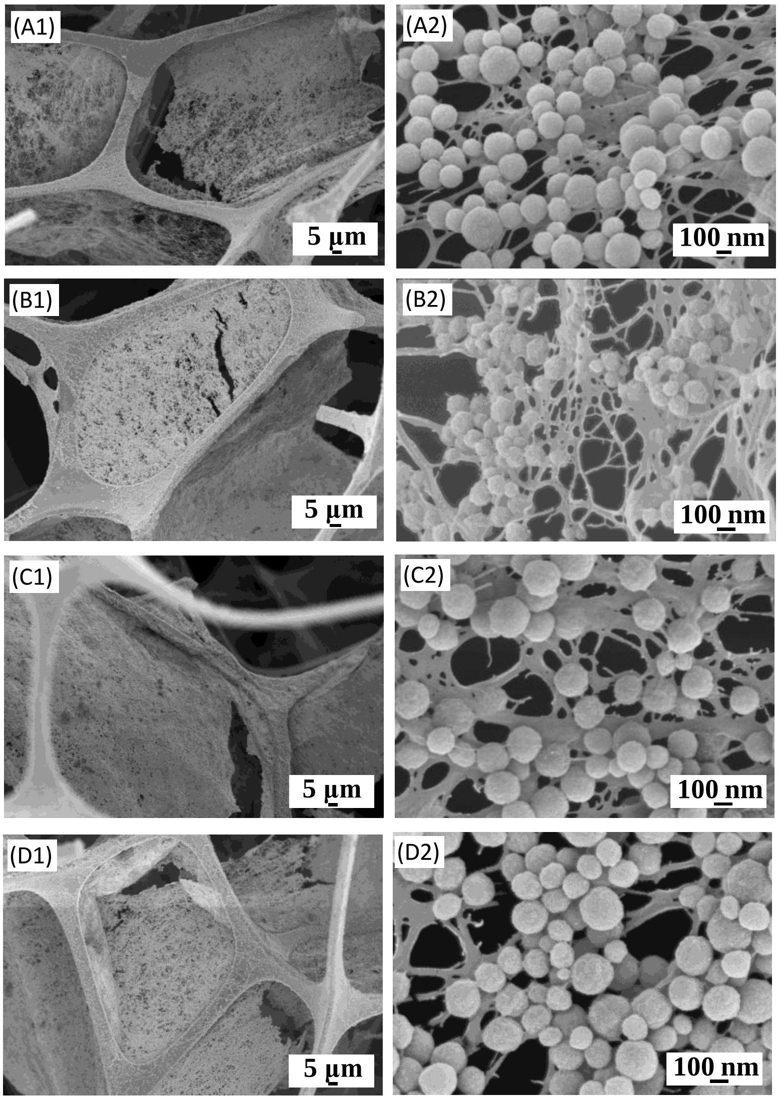

Figure S7. SEM images of of the MOF-F@MS after the rigorous treatments of A) vortex, B) pressing, C) soaking and D) ultrasound. 1 and 2 present the same sample under different magnification scales.

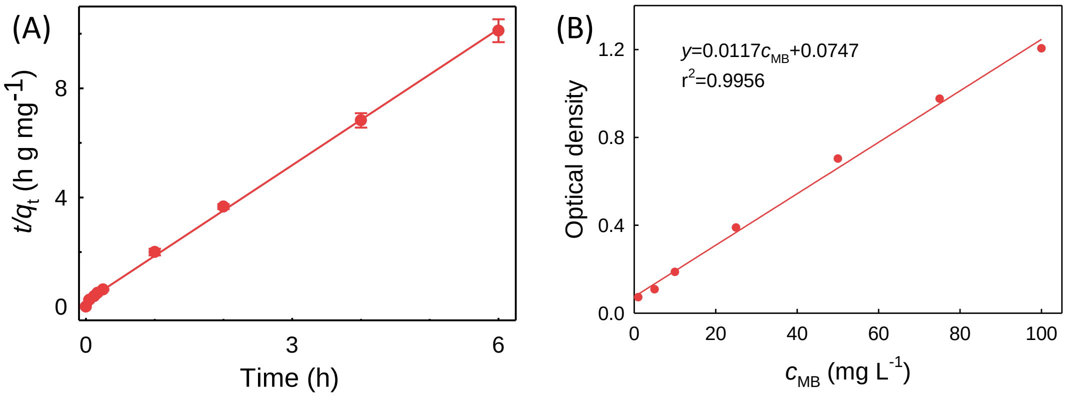

Figure S8. A) Pseudo-second-order kinetics curve. B) Calibration curve for the quantification of MB.

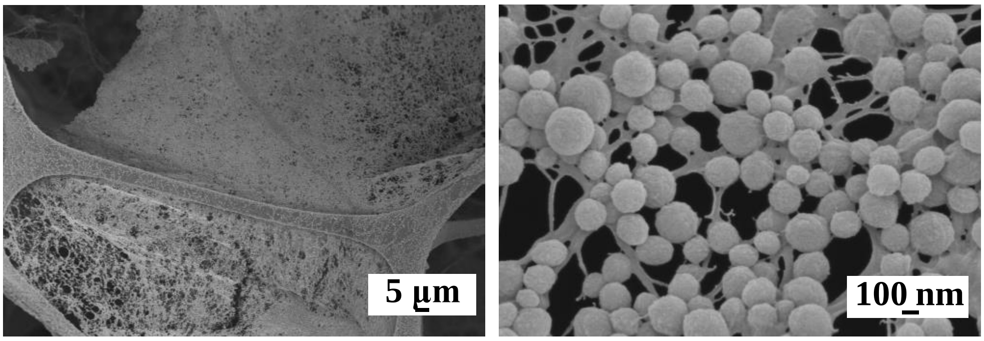

Figure S9. SEM images of the MOF-F@MS after the 6-h adsorption of methylene blue with different magnification scales.

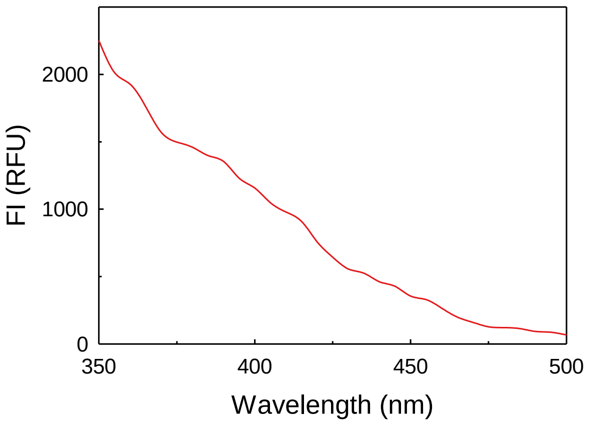

Figure S10. Fluorescent emission spectra of the solution after the 24-h immersion of MOF-F@MS in water.

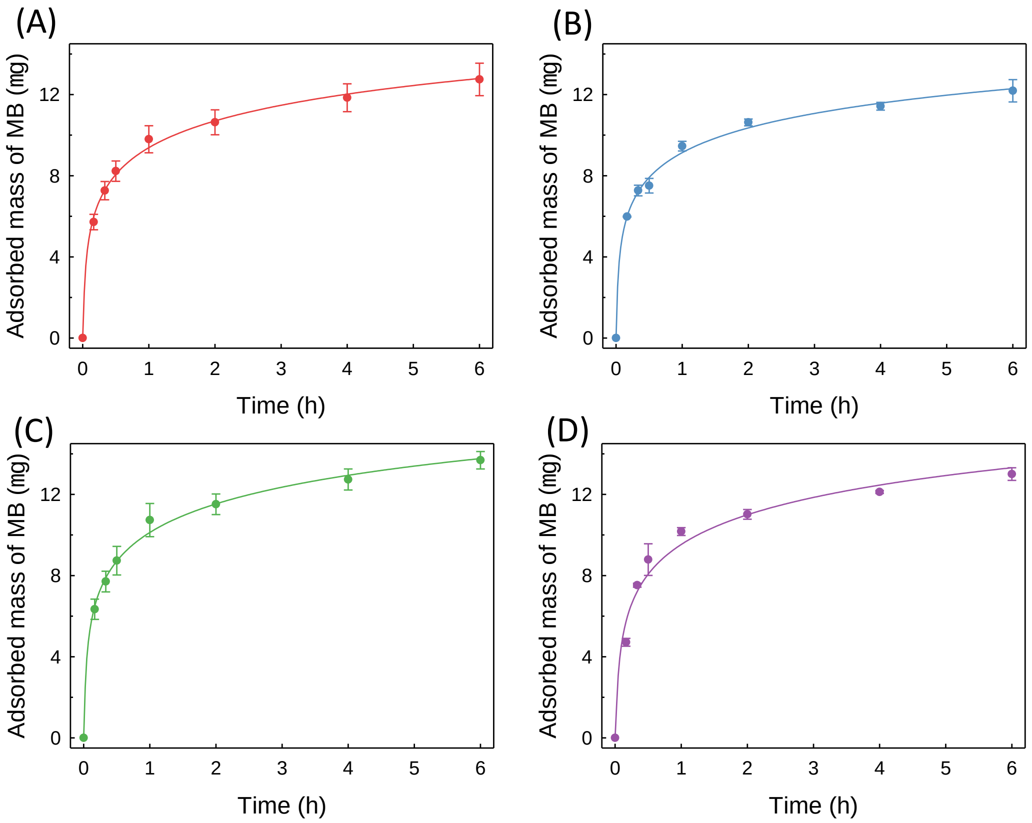

Figure S11. Time-dependent adsorption curves of the MOF-F@MS samples after the rigorous treatments of A) vortex, B) pressing, C) soaking and D) ultrasound towards MB. For each condition, the experiment was repeated on three samples.

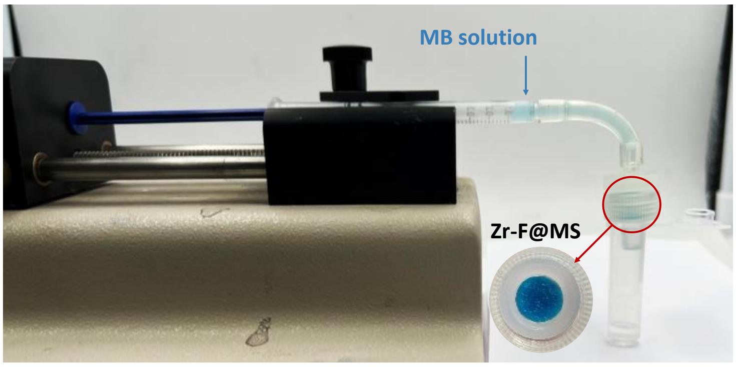

Figure S12. The photograph of a home-made Zr-F@MS-based filtration system for the dynamic adsorption of MB.

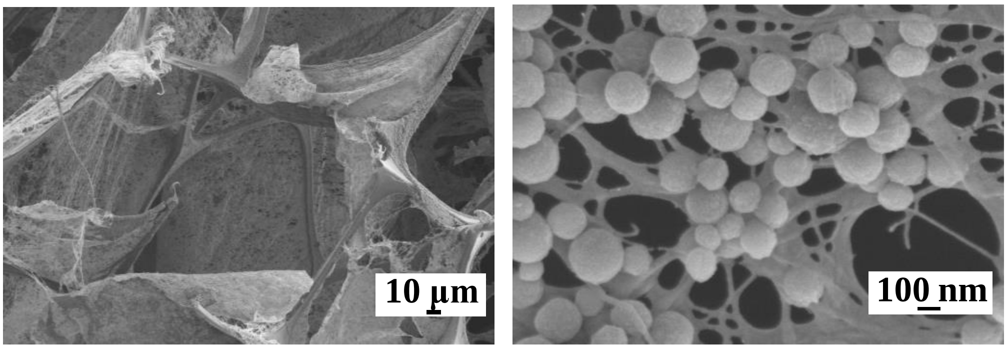

Figure S13. SEM images of the MOF-F@MS after 20 times filtration with different magnification scales.

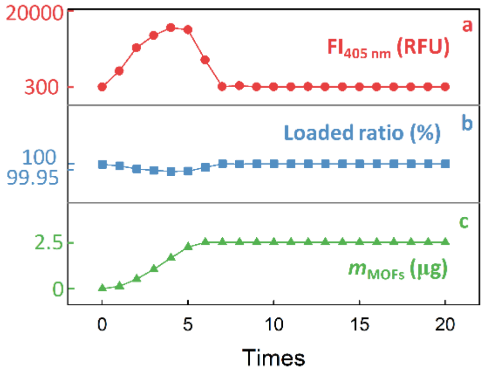

Figure S14. Characterization of the detachment of Zr-LMOFs from MOF-F@MS during the 20 times filtration (to avoid fluorescent quench caused by MB, 400 µL water was used as the target solution for one time filtration). Line a is the FI at 405 nm of the filtrate. Line b presents the loaded ratio of Zr-LMOFs in MOF-F@MS in each filtration test. Line c presents the accumulated mass of the detached Zr-LMOFs.

The fluorescence intensity of the solution at 405 nm increased in the first 6 rounds filtration, however, which corresponds to a really low loss ratio of 0.25%. Then, the fluorescence kept almost unchanged with a negligible value around 300 (the value is 283 ± 5 for water as comparison). This indicated that a few loosely loaded Zr-LMOFs would be detached at the beginning but the most others remained stable in the MOF-F@MS, confirming the structural reliability of MOF-F@MS for dynamic adsorption. For practical application, the MOF-F@MS could be rinsed for several rounds to avoid the interference of loosely loaded MOFs.

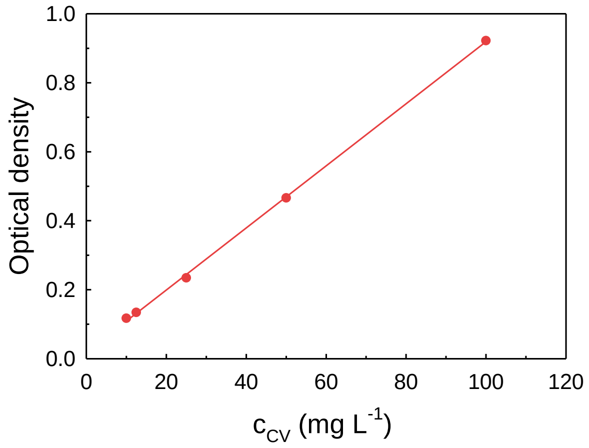

Figure S15. Calibration curve for the quantification of CV.

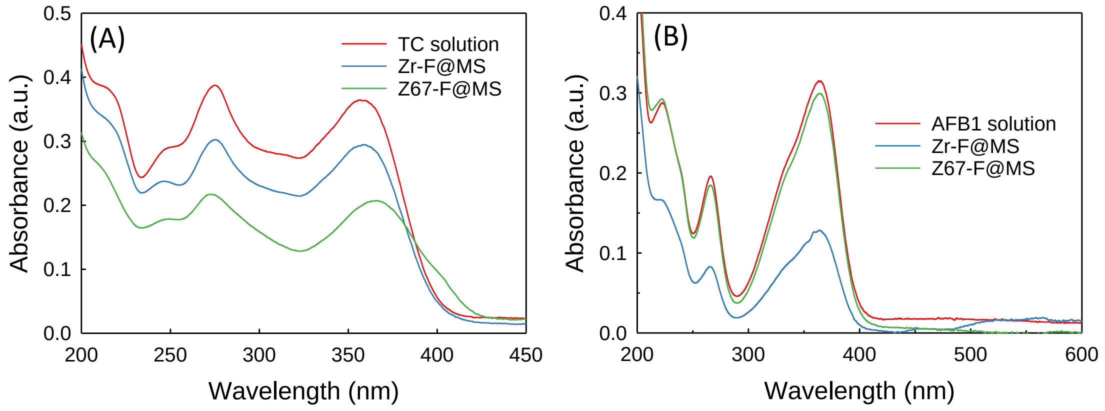

Figure S16. UV-Vis spectra of A) TC and B) AFB1 solution before and after the incubation within Zr-F@MS and Z67-F@MS.

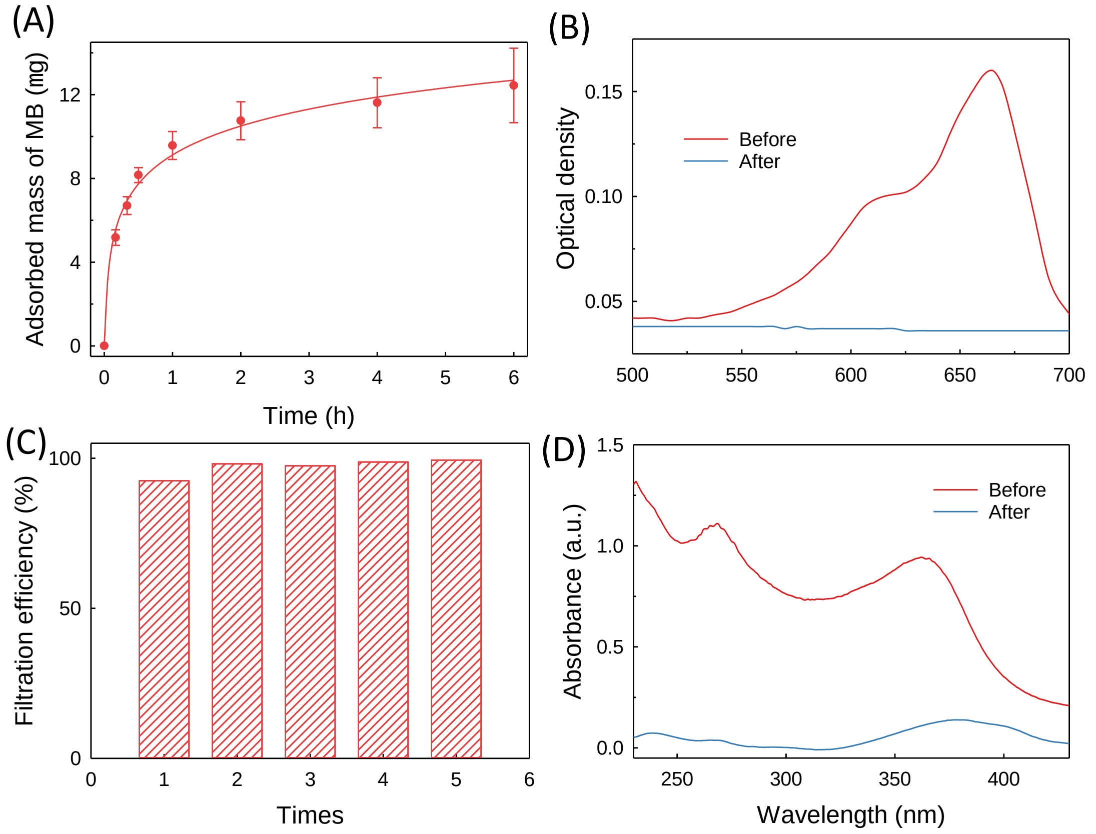

Figure S17. Practicality of the MHPMs for the adsorption of targets in real-world samples. A) Time-dependent adsorption curve of MOF-F@MS towards methylene blue added in river water (50 mg L^{-1}, 400 μL). B) UV-Vis spectra of the methylene-blue-added river water sample (10 mg L^{-1}, 400 μL) before and after the filtration through MOF-F@MS, and C) the corresponding filtration efficiency for each filtration test. D) UV-Vis spectra of the mixture solution of TC (16.7 mg L^{-1}) and AFB1 (16.7 mg L^{-1}) added in feed sample before and after the adsorption of Zr-Z67-F@MS.

References

[1] F. Han, X. Qi, L. Li, L. Bu, Y. Fu, Q. Xie, M. Guo, Y. Li, Y. Ying, S. Yao, *Adv. Funct. Mater.* **2014**, *24*, 5011-5018.
